# An Uncommon Presentation of a Common Disease: A Review of Gastric Metastasis From Breast Carcinoma

**DOI:** 10.7759/cureus.11920

**Published:** 2020-12-05

**Authors:** Luis Geada, Micaella Kantor, Karthik Mohan, Daniel Weingrad, Luis S Nasiff

**Affiliations:** 1 Department of General Surgery, Aventura Hospital and Medical Center, Miami, USA; 2 Department of General Surgery, Kendall Regional Medical Center, Miami, USA; 3 Department of Gastroenterology, Palm Springs Hospital, Miami, USA; 4 Department of Gastroenterology, Palmetto General Hospital, Miami, USA; 5 Department of Surgical Oncology, Aventura Hospital and Medical Center, Miami, USA

**Keywords:** breast cancer management, gastric malignancy, breast cancer metastasis, breast cancer

## Abstract

Breast cancer is the most common cancer in women, and the leading cause of cancer-related deaths worldwide. Despite advances in screening and treatment modalities, distant metastasis still develops. Breast cancer metastasis to the gastrointestinal tract is very rare, therefore, its diagnosis, therapeutic strategies, and prognosis pose a clinical problem for clinicians. We summarize the current knowledge regarding the clinicopathological characteristics and diagnostic strategies for metastatic tumors in the stomach of breast origin.

## Introduction and background

Breast carcinoma is the most common malignancy in women accounting for 25.1% of all cancers in women and the leading cause of cancer-related death among females worldwide [1]. Despite early recognition by screening and efficacy of new treatment modalities, many patients eventually develop metastatic disease either by locoregional recurrence or distant metastases. The most common sites of metastases include the skeleton, lungs, brain, and liver [2-5]. Metastatic disease to the gastrointestinal tract is rare and poses a clinical problem in both diagnosis and management. Primary malignancies that most commonly metastasize to the stomach include breast cancer (27.9%), lung cancer (23.8%), esophageal carcinoma (19.1%), renal cell carcinoma (7.6%), and malignant melanoma (7.0%) [6-8]. Aside from melanoma, which has an unusual predilection for metastasis to the gastrointestinal tract, the other primary sites generally mirror their incidence in the general population [8-10].

Based on clinical and autopsy findings, the reported incidence of gastric metastasis is 0.2%-0.7% [6,7,11]. The postmortem frequency of gastric metastasis from breast carcinoma is estimated to be at 0.8%-18%. The majority of cases originate from invasive lobular carcinomas (ILCs) [2,12-15]. 

Only a few cases of metastasis from breast cancer to the gastrum have been noted because of its low incidence. There is little documented on the characteristics, outcomes, and endoscopic findings on metastasis of ILCs to the stomach. The factors involved in prognosis and the particular treatments for these patients needs further investigation because of this [8].

## Review

The gastrointestinal tract is a very uncommon site for breast cancer metastasis. While invasive ductal carcinoma (IDC) represents about 80% of all breast carcinomas, the majority of gastric metastasis arise from ILCs (65.4%) and only 24.4% from IDC [16-22]. In the majority of the cases, the diagnosis of breast cancer precedes the signs and symptoms of metastatic disease [1,23]. It has been estimated that the mean time from the diagnosis of breast cancer to reported metastasis is six to seven years. However, it has been reported as long as 20 to 30 years later, even after surgical resection [4,14,24,25]. Disseminated disease is seen in 90%-94% of patients at the time of diagnosis of gastric metastasis. Concomitant metastatic sites commonly include bone (60%), followed by liver (20%) and lung (18%) [12,26-28]. After the stomach, other intra-abdominal organs involved, in the order of increasing rarity, include the colon, rectum, and small bowel [17]. Gastric metastasis may also present as primary or peritoneal carcinomatosis, involving the peritoneum, adrenals, and lung pleura [17,23]. There are only a handful of case reports in which gastric metastasis was diagnosed prior to or at the same time as breast carcinoma.

Patients present with a landscape of nonspecific symptoms such as anorexia, dysphagia, early satiety, postprandial bloating, epigastric pain, melena, nausea, and vomiting [12,24,29]. Aside from symptoms being nonspecific, they also mimic the side effects of chemotherapy or other medication, liver metastasis, and even hypercalcemia of malignancy [2]. This can further cause a delay in diagnosis or misdiagnosis of the disease. Clinically and endoscopically, it is almost impossible to differentiate primary GI tumors or non-Hodgkin lymphoma from metastatic disease [12,29].

The endoscopic features can range from benign-appearing lesions (such as gastritis) to diffuse or ulcerated tumors (mimicking primary gastric carcinoma or lymphoma) [12,27,30]. Currently, there are three main categories of lesions described: localized tumor deposition (18%), diffuse infiltration (i.e. linitis plastica type or gastritis) (57%-73%) and external compression (25%) [2,12,15,27]. The most common presentation is the diffuse type- linitis plastica [15,27,30]. This lobular breast cancer-induced syndrome was first described by Cormier et al. in 1980 [26]. It resembles a Borrmann Type 4 advanced gastric cancer with diffuse hypertrophy and sclerosis of the gastric mucosal folds and deep invasion of the submucosal and muscularis propria layers [30-34]. Localized infiltration presents macroscopically with either large ulcers, nodules, or polypoid mass lesions [17,31]. Though infrequent, polypoid lesions may grow large and resemble gastrointestinal stromal tumors [23,33]. These localized lesions are often described as “bull’s eye” or “target” lesions because the submucosal tumors (SMT) tend to have a central depression covered by an intact mucosa or a raised SMT with an ulcerated center [6,30,34-36]. Interestingly, localized-type nodular lesions are more commonly seen in metastatic ductal-type breast carcinoma [21,28]. External compression can occur when a ring-like tumor forms around the cardia or pylorus, for instance. This may lead to pseudoachalasia or gastric outlet obstruction [6,35,37,38]. Very rarely, metastatic infiltration of the duodenum or distal bile ducts has been reported which may present with jaundice [23,33].

Because external metastatic invasion of the stomach often spares the superficial mucosal layer, endoscopic biopsies have an increased false-negative rate [17,34,39]. In gastric metastasis secondary to ILC, initial superficial biopsies were reported as normal in up to 46%-50% of the cases because the invasion was limited to the submucosa and seromuscular layers [23,24]. If there is a high index of suspicion, non-conventional techniques such as macro-biopsies or endoscopic ultrasound-guided fine-needle aspiration cytology should be used whenever possible [14,40]. Morphologically, the infiltrated gastric tissue commonly shows poorly cohesive, round tumor cells with an occasional intracytoplasmic lumen arranged in linear cords between the normal gastric glands [17,41]. These cells resemble signet-ring cell tumors in the WHO classification. It has been suggested that a well-defined univacuolated cytoplasm is relatively specific for ILC metastasis [15,42]. Whereas, multivacuolated cytoplasm suggests a primary gastric signet ring cell carcinoma. However, some data is still equivocal, requiring further investigation [15,42]. Commonly, biopsy specimens are also misinterpreted as poorly differentiated gastric carcinoma [15]. Other conditions that mimic signet-ring carcinoma include lymphomas, macrophages, xanthomas, or even gastritis [15,42]. Keratin stains are therefore required to diagnose the presence of invasive tumor cells [17].

In order to obtain the definitive diagnosis of metastatic gastric cancer, immunoprofiling of the tumor cells is imperative. Specific biological immunohistochemical markers for breast, as well as other organs, can aid in the differential diagnosis of neoplasms. Among these, estrogen receptor protein (ER) is the most influential and well-known marker for differentiating metastatic breast cancer. ER is expressed in 72%-90% of breast tumor cells. Progesterone receptor protein (PR) is seen in approximately 33% of cases [27,30,34,42]. However, testing for ER and PR biomarkers alone is not suitable because not all breast cancer cells express these hormone receptors. It has also been widely reported that the receptor expression of the primary breast cancer may be lost in metastatic tissue due to disease progression or following prolonged hormonal therapy [43-45]. In addition, various studies have shown ER and/or PR positivity within primary gastric cells in up to 28% and 12% of the cases, respectively [34,46-48]. Velthuysen et al. [44] demonstrated that second-generation antibodies against ER-alpha can be more specific and reliable for determining breast origin, as they are not expressed by gastric tumor cells. However, ER-alpha is limited in those with ER-negative breast cancer. The human epidermal growth factor receptor 2 (HER-2) is dysregulated in numerous types of solid tumors including those of breast and gastric origin [43,45]. Therefore, its role in diagnosis is also limited and is useful only when tailoring treatment [8].

Other breast specific markers include gross cystic disease fluid protein (GCDFP-15), mammaglobin, and GATA protein type 3 [49]. GCDFP-15 is a pathological secretin released by the breast particularly in the setting of apocrine metaplasia of the breast [49]. It is highly specific for mammary differentiation and yields a specificity of 93%-100% with a sensitivity of 11%-76%. It has the highest expression in the lobular carcinoma subtype [24,30,45]. Mammaglobin, a mammary gland-specific gene that is overexpressed in breast cancer, has an expression rate of 47.8% to 80% in primary and metastatic breast carcinomas [34]. GATA protein type 3 is a multifunctional transcription factor and part of the GATA family of zinc fingers DNA binding proteins. It is only present in breast and urothelial carcinomas [30]. It is expressed in up to 96.6% of ER-positive breast carcinomas, however, its expression decreases to 21.6% in triple-negative cases [15,24].

Cytokeratin stains, specifically CK5/6, CK 7, and CK 20, have also been used to aid in differentiating metastatic gastric tumors [15]. CK7+/CK20- are usually expressed in breast, ovarian, and lung adenocarcinomas, however, it can be seen in up to 33% of gastric adenocarcinoma cases [15,24]. CK7-/CK20+ and CK20+/ER- are patterns typically seen in primary gastric carcinomas [15,24]. CK7 alone has no discriminatory value because it is present in 71% of gastrointestinal malignancies and 95% in lobular carcinomas [5]. Positive CK5/6 is present in 61% of breast cancers and in 16% of gastric cancers [14]. In a large retrospective study that evaluated a broad panel of immunohistochemical stains, O’Connell et al. [45] found CK20+, MUC6, DAS-1 (a monoclonal antibody), and CDX2 (a caudal type homeobox transcription factor) to be 100% specific for primary gastric carcinomas.

The loss of E-cadherin expression occurs in both invasive lobular carcinoma of the breast and in diffuse-type or poorly cohesive gastric carcinoma [34,44]. Mutations in the CDH1 gene leads to E-cadherin inactivation and subsequently may lead to a familial cancer disorder called hereditary diffuse gastric cancer (HDGC) (12). Patients with HDGC have a 56%-70% lifetime risk of developing gastric cancer, and in women, a 42% lifetime risk of developing lobular carcinoma [12]. Mutations in E-cadherin lead to impaired tumor suppression and cell adhesion which increases the likelihood of tumor cell invasion and metastases. It has been reported that 85% of ILCs and 50% of gastric carcinomas have decreased E-cadherin immunoreactivity [44]. Therefore, in the absence of E-cadherin expression, one must differentiate between the presence of two synchronous neoplasia or primary neoplasia with metastases. Even though the exact mechanism regarding the different metastatic patterns between ILCs and IDCs remains obscure, the loss of E-cadherin expression may play a role in the more diffuse metastatic growth pattern of ILCs.

Comparison of endoscopic biopsies with the prior or with the present breast carcinoma specimen improves diagnosis. However, antigen expression may differ between primary and metastatic tumor sites [2,6,45]. In numerous studies, patients were diagnosed with metastatic breast carcinomas only after a gastrectomy was performed for primary gastric carcinoma [15,42]. Taken together, relying solely on tumor biomarkers for accurate diagnosis may be vexing given the lack of a specific immunohistochemistry biomarker for breast cancer plus the instability of the tumor phenotype along the disease progression.

It is crucial to distinguish metastatic gastric tumor from primary gastric carcinoma because treatment and prognosis greatly differ between the two. Only a small number of studies have investigated the prognostic factors and standard treatment strategies for gastric metastasis from breast carcinoma because of its low incidence. The presence of gastric metastases reflects advanced disease which makes prognosis poor. Following the diagnosis of gastric metastasis, the median survival rate is estimated to be between 10-28 months [17,27,19,42]. Survival is further decreased in those with multiorgan metastasis.

In patients with primary gastric cancer, without distant or peritoneal metastasis, surgical resection is the most effective treatment [34]. Because gastric metastasis from breast cancer is a systemic disease, metastatic gastric cancer is treated systemically with chemotherapy, hormone therapy, or combined therapy. Therefore, hormone receptor status is crucial. The treatment plan is often individualized and based on age, clinical condition, hormone receptor status, and previous treatment [2]. Patients who present with linitis plastic type metastasis and are hormone receptor positive have been shown to respond well to conventional chemotherapy and hormone therapy [19,27]. In two large retrospective studies, remission rates were achieved in 46% to 52% of the time. This prolonged survival time by two to three years [2,7]. Zelek et al. [50] reported a median survival of 21 months in patients who received chemotherapy. They compared this to less than 12 months in those who underwent surgical gastric resection. Surgical intervention did not significantly improve survival (28 vs. 26 months). However, in patients with exclusive gastric metastasis that underwent palliative surgical resection, median survival was prolonged (44 vs 9 months). Unfortunately, this was not statistically significant. Surgical intervention is reserved for patients with unique localized gastric metastasis and palliatively in those with complications such as intestinal obstruction or bleeding [30,34,50]. Minimally invasive interventions, such as placement of endoscopic self-expandable metallic stents (SEMS) are being further investigated and used for symptomatic management, for instance, in gastric outlet obstruction caused by metastatic gastric tumor [34].

## Conclusions

To this day, the literature published on metastatic tumors of the stomach is still incomplete, the majority being case reports and few small retrospective studies. Breast cancer metastasis to the gastrointestinal tract, particularly the stomach, can present with a wide range of clinical and radiological findings, often mimicking other more common disorders. Due to the high incidence of breast carcinoma, clinicians should be aware of the possibility of gastric metastasis in an adequate clinical setting. Prompting early investigation can prevent disease progression and complications. Diagnosis poses many challenges, particularly in cases such as ours, where symptoms of gastric metastasis precede the diagnosis of breast carcinoma. Large prospective studies are needed to improve the understanding of the biological and pathological characteristics of these tumors since clinical presentation is unspecific and endoscopic appearance is heterogeneous. The mechanism underlying the metastatic pattern of ILCs is still unclear, however, the loss of expression of E-cadherin, the cell-cell adhesion molecule, in this subtype may contribute to its particular pattern of spread. We believe understanding the specific biological and molecular mechanisms of ILCs will contribute to advances in the development of more specific therapeutic approaches, thus decreasing the rate of metastasis spread. Therefore, there is an urgent need for further investigation on the biological and clinical behavior of breast carcinomas, particularly ILCs given their more aggressive comportment and metastasis to unusual organs, such as the gastrointestinal tract.
